# Willis covered stent treatment for internal carotid artery pseudoaneurysm: A meta-analysis of efficacy and safety

**DOI:** 10.1016/j.heliyon.2023.e23022

**Published:** 2023-11-29

**Authors:** Jiahe Tan, Rui Song, Siyue Luo, Yinrui Ma, Jun Su, Baoping Qin, Zhaohui He

**Affiliations:** a Department of Neurosurgery, First Affiliated Hospital of Chongqing Medical University, Chongqing, 400016, China; b Department of Infectious Diseases, Second Affiliated Hospital of Chongqing Medical University, Chongqing, 401336, China; c Clinical Medicine, Second Clinical College of Chongqing Medical University, Chongqing, 400042, China; d Department of Neurosurgery, People's Hospital of Shi Zhu Tu Jia Zu Autonomous County, Chongqing, 409100, China

**Keywords:** Willis covered stent, Pseudoaneurysm, Efficacy, Safety, Meta-analysis

## Abstract

**Background:**

Pseudoaneurysm (PSA) of internal carotid artery is a rare but severe cerebrovascular disease and difficult to repair surgically. A novel medical device called Willis covered stent (WCS) has been created especially for the treatment of complex cerebrovascular diseases. However, the efficacy and safety of WCS therapy for PSA are still debatable. Additional substantial proof is needed.

**Methods:**

To find research pertaining to WCS treatment for PSA, a systematic review of literature was conducted in the Medline, Embase, Web of Science, CNKI, Wanfang, and CBM databases. The results comprising the data of intraoperative situation, postoperative situation, and follow-up were then included in a meta-analysis.

**Results:**

The criteria were met by 11 noncomparative studies with 152 patients and 157 PSAs. Technical success rate was nearly 100 % (>0.999 (95 % confidence interval (CI), 0.958, 1.000)), complete occlusion rate was 97.8 % (95 % CI, 0.932, 1.000), and side branch occlusion rate was 0.5 % (95 % CI, 0.001, 0.045). The rates of acute in-stent thrombosis (<0.001 (95 % CI, 0.000, 0.013)) and hemorrhage (<0.001 (95 % CI, 0.000, 0.005)) were both less than 0.1 %. In postoperative situation, surgery-related mortality rate was less than 0.1 % (<0.001 (95 % CI, 0.000, 0.005)). The rates of recurrence (<0.001 (95 % CI, 0.000, 0.002)) and parent artery stenosis (<0.001 (95 % CI, 0.000, 0.008)) were both less than 0.1 %, while late in-stent stenosis occurred in 1.3 % (95 % CI, 0.000, 0.053) of patients. In the end, 98.5 % (95 % CI, 0.943, 1.000) of patients had a good outcome.

**Conclusion:**

The application of WCS could be effective and safe for PSAs. The findings of this study could serve as a reference for upcoming clinical trials.

## 1 Introduction

Pseudoaneurysm (PSA) of internal carotid artery (ICA), which is commonly caused by trauma and iatrogenic parameters, is a rare but severe cerebrovascular disease. It accounts for only 1 % of intracranial aneurysms, but it has a mortality rate of 50 % or higher after rupturing [1]. The natural history of PSA is still not well-understood. According to animal model, the formation of traumatic PSA is divided into four stages: the injury of artery and formation of hematoma (within 3 days), the early stage of PSA formation (4–10 days), the stage of PSA formation (11–30 days), and the enlargement, rupture and bleeding of PSA (30–40 days) [2]. After a completely damage of the arterial wall, a hematoma may form around the rupture point of the artery due to the thick soft tissue around the artery. Then because of the continuous impact force of arterial pulsation, the hematoma can communicate with the ruptured artery, which leads to a pulsating hematoma. The hematoma cavity will gradually liquefy, and the fibrous tissue envelope will gradually form around the hematoma, and this condition eventually leads to a PSA [3]. Unlike a true aneurysm, a PSA does not have the three layers of the outer membrane, the middle elastic fiber and the inner membrane of the artery. These pathophysiological processes impart friability, lack of true neck, and fusiform morphology to PSA, in addition to its short natural history and high mortality rate after rupture, making the treatment of PSA extremely challenging [4].

Unfortunately, as far as we know, there are no clinical guidelines of PSA treatment. Endovascular therapy has become a more effective and dependable treatment for PSA owing to advancements in procedures and the creation of novel materials, whereas microsurgery is typically only used when endovascular therapy fails [5]. In recent years, the concept “repairment of parent artery” gradually replaces “embolization of PSA” due to the emergence of flow diverter (FD) and covered stent [6]. That is, most clinical centers have reached a consensus that repairment of parent artery using FD or covered stent by endovascular therapy should be the first choice to treat PSAs. However, the optimal management is still much debated.

A novel device known as Willis covered stent (WCS) (MicroPort, Shanghai, China) has been developed and used in China to treat intracranial complicated vascular lesions. It is made up of a low pressure and elastic balloon catheter, an expended polytetrafluoroethylene membrane, and a bare stent [7]. This “China Option” fits the concept of parent artery repairment, and avoids the occupied effect of traditional therapies. Besides, the placement of WCS is simple, so the operation time is short. What's more, WCS can immediately isolate PSA cavity and parent artery, exclude PSA from the circulation, avoid the occurrence of thrombosis in the PSA cavity to the maximum extent, maintain the patency of parent artery, eliminate the pathological injury of artery endothelium, and return the parent artery's passage and hemodynamics to normal; thus, it can realize vascular reconstruction and treatment of PSA [8]. These properties and purported benefits of WCS make it uniquely suited for treating PSA.

Several clinical centers in China have applied WCSs to treat PSAs, but the efficacy and safety were unknown as a result of the disparate findings of these studies of small sample size and due to the rarity of PSA and the innovation of WCS. A higher level of evidence is required. Thus, we aim to systematically review studies pertaining to WCS treatment for PSA and conduct a meta-analysis to include the results of efficacy and safety.

## 2 Methods

The review was prospectively registered with the PROSPERO database (CRD42022380183). And this research was conducted by following the guidelines of the Preferred Reporting Items for Systematic reviews and Meta- Analyses (PRISMA) and the guidelines of the Assessing the methodological quality of systematic reviews (AMSTAR) [9,10].

### 2.1 Literature search

A thorough literature search in the Medline, Embase, Web of Science, CNKI, Wanfang, and CBM databases obtained studies pertaining to WCS treatment for PSA. Search terms included “pseudoaneurysm,” “false aneurysm,” “aneurysm, false,” “Willis stent,” and “Willis covered stent” in “AND” and “OR” combinations. The time for literature search was ended on November 23, 2022. Only English and Chinese were available as publishing languages. No restriction was applied on the publishing year.

### 2.2 Inclusion and exclusion criteria

The conditions for inclusion were as follows: (1) Noncomparative studies of PSA treatment with WCS. (2) A detailed description of PSA. (3) WCS was treated by skilled surgeons or interventional radiologists, and perioperative antiplatelet therapy must follow a standard protocol. (4) Sample size of a single study was more than 2 patients. (5) Initial data of the outcomes were reported. (6) Studies with moderate or high quality (≥4 points). The conditions for exclusion were as follows: (1) Repetitive studies. (2) Other types of studies in addition to noncomparative study. (3) PSA co-treatment. (4) An absence of raw data. (5) Studies with low quality (≤3 points). In addition to the abovementioned criteria, the most recent and comprehensive research was retained while duplicate cohort studies were excluded. Two authors (Tan and Song) independently selected studies based on the aforementioned criteria. If any disagreements would arise, then the two authors would discuss with a third author (Luo) to reach a consensus.

### 2.3 Data extraction

Date included baseline characteristics: first author, publication year, clinical center, number of patients, sex, age, number, status, size, neck width, and location of PSAs. Intraoperative situation: number of WCSs per patient, technical success, complete occlusion, side branch occlusion, acute in-stent thrombosis, and hemorrhage. Postoperative situation: surgery-related mortality. Follow-up data: duration, recurrence, parent artery stenosis, late in-stent stenosis, and good outcome. The sample size of a single study was the number of patients in the baseline characteristics of the study, and the overall sample size depended on the total number of patients in all studies eventually included. Technical success was described as WCS being implanted successfully into the parent artery of PSA. Characteristics of PSAs, occlusion, thrombosis, hemorrhage, recurrence, and stenosis were confirmed by imaging examination. The location of PSAs was distinguished by the Bouthillier segmental method of the ICA. Other factors considered were the epistaxis or intracranial hemorrhage stopped and no longer relapsed, focal neurological deficits such as the vision loss and headache improved or stopped getting worse, and a modified Rankin Scale score between 0 and 2 indicating a good outcome. Two authors (Tan and Song) independently extracted the data. If any disagreements would arise, then the two authors would discuss with a third author (Luo) to reach a consensus.

### 2.4 Quality assessment

Methodological integrity of each study was assessed using the 11-item checklist of the Agency for Healthcare Research and Quality (AHRQ) [11]. One point was given for “YES” of each above item, while “NO” or “UNCLEAR” got no point. The studies were graded as having low quality (≤3 points), moderate quality (4–7 points), and high quality (≥8 points). Two authors (Tan and Song) independently assessed the quality. If any disagreements would arise, then the two authors would discuss with a third author (Luo) to reach a consensus.

### 2.5 Statistical analysis

Data management, the transformation of the effect size, calculation of the pooled risk difference, and corresponding 95 % confidence interval (CI) were performed using the ‘metaprop’ code in the Stata statistical software (version 16.0) [12]. A random-effect model was selected after considering data compatibility to obtain each event's rate. I-squared (I^2^), Tau-squared (Tau^2^), Chi-squared (Chi^2^) and its corresponding P value were used to measure the heterogeneity between the studies. Whether the significance of heterogeneity was evaluated by the three indicators together. I^2^ ≥ 50 % indicated significant heterogeneity, Tau^2^ > 0 indicated heterogeneity, and P value of Chi^2^ < 0.1 indicated significant heterogeneity [13,14]. Forest plots were generated to display the results graphically. Publication bias test and sensitivity analysis were not completed as limited by the nature of noncomparative study [15].

## 3 Results

### 3.1 Literature search

**Fig. 1:**
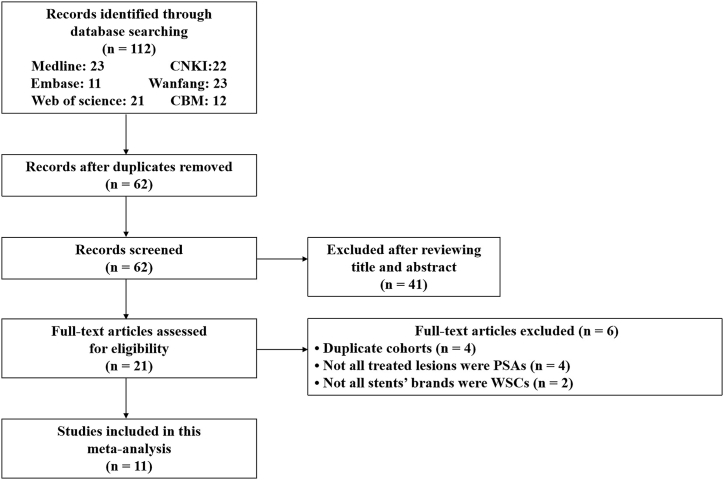
Flowchart of the literature search performed. PSA, pseudoaneurysm; WCS, Willis Covered Stent.

A thorough search of the literature produced 112 records. A total of 62 records were left after duplicate entries were removed by the title and abstract review. Then, 21 of them were left for the full-text review. Thereafter, 4 were excluded because of duplicate cohorts, 4 were excluded because not all treated lesions were PSAs, and 2 were excluded because not all stents’ brands were WCSs. Ultimately, 11 noncomparative studies were included in our research [[16], [17], [18], [19], [20], [21], [22], [23], [24], [25], [26]]. A flow diagram is shown in Fig. 1.

### 3.2 Characteristics of included studies

In the studies published from 2011 to 2022, the total sample size was found to be 152 patients (106 men and 46 women) with 157 PSAs. They were from 12.0 to 51.3 years old on average. All included studies were conducted at Grade 3A hospitals in China (top clinical centers in China). One was a multicenter study and the rest were single center studies. All included studies provided thorough descriptions of the data of intraoperative situation, postoperative situation, and follow-up. Unfortunately, characteristics of PSAs were not reported in some works. The characteristics and outcomes of each research are summarized in Table 1, Table 2.

**Table 1:** Characteristics of the included studies.

Author,y	Patients,n	Males/Females,n	Age,y	PSA Status, n	PSA Size, mm	PSA Location, n
Wang2011	13	12/1	mean 34.4 ± 13.6	unruptured: 7	mean 11.8 ± 7.3 (2.5–30.0)	C2: 1
			11–60	ruptured: 6		C4: 5
						C5: 1
						C6: 3
						C7: 3
Pan2015	13	11/2	mean 25.8 ± 9.5	NA: 15	NA	NA
			6–38			
Liu2016	3	1/2	mean 12 ± 4.2	ruptured: 3	NA	C2: 1
			6–15	NA: 1		C6: 1
						C7: 2
Kang2019	5	3/2	median 54.0	unruptured: 5	NA	C2: 5
			38–67			
Liu2019	3	1/2	mean 51.3 ± 22.3	unruptured: 2	NA	NA
			22–76	rupture: 1		
Deng2020	5	2/3	mean 44.8 ± 11.6	ruptured: 6	NA	C4: 5
			31–62			C7: 1
Liao2020	17	14/3	mean 33.1 ± 14.1	unruptured: 17	5.0–20.0	C4: 15
			19–45			C5: 2
Wangkai2020	3	2/1	mean 35 ± 16.1	NA: 3	mean 10.9 ± 2.9 (8.3–15.0)	C4: 1
			16–56		mean neck width 3.5 ± 1.1 (2.1–4.8)	C6: 1
						C7: 1
Wangwei2020	19	14/5	mean 30.9 ± 12.6	NA: 20	median 5.0 (2.0–40.0)	C4: 16
			11–63		median neck width 3.0 (2.0–9.0)	C5: 2
						C6: 2
Zhao2021	15	13/2	mean 39.0 ± 10.7	unruptured: 4	mean 7.5 ± 2.2 (1.7–16.0)	C3: 6
			15–51	ruptured: 11	mean neck width 3.5 ± 1.9 (0.5–7.7)	C6: 4
						C7: 5
Lu2022	56	33/23	mean 46.7 ± 13.0	unruptured: 35	<10: 23	C2: 10
				ruptured: 21	10-25: 25	C4: 20
					>25: 8	C5: 5
					wide-necked: 20	C6: 21

y, year; n, number; PSA, pseudoaneurysm; mm, millimeter; NA, not available.

**Table 2:** Outcomes of the included studies.

Author, y	WCSs per Patient, n	Technical Success, n	Complete Occlusion, n	Side Branch Occlusion, n	Acute In-stent Thrombosis, n	Intraoperative Hemorrhage, n	Surgery -related Mortality, n	Duration, m	Recurrence, n	Parent Artery Stenosis, n	Late In-stent Stenosis, n	Good Outcome, n
Wang 2011	1 device: 12 2 devices: 1	13	9	0	0	0	0	mean 14.8 ± 8.6 3–36	1	0	0	12/13
Pan 2015	1 device: 13	12	12	0	0	0	0	mean 22.6 ± 12.7 3–48	0	0	0	12/12
Liu 2016	1 device: 2 2 devices: 1	3	3	0	0	0	0	mean 10.0 ± 5.1 3–15	0	0	0	3/3
Kang 2019	1 device: 5	5	5	0	0	0	0	3	0	0	0	5/5
Liu 2019	1 device: 3	3	3	0	0	0	0	mean 8.3 ± 5.0	0	0	0	3/3
Deng 2020	1 device: 4 2 devices: 1	5	5	0	0	1	1	6–12	0	0	0	4/4
Liao 2020	1 device: 17	17	17	0	0	1	1	mean 12.5 8–24	0	1	0	15/16
Wangkai2020	1 device: 3	3	3	0	0	0	0	mean 44.0 ± 7.0 36–53	0	0	0	3/3
Wangwei2020	1 device: 19	19	18	0	1	0	0	mean 9.0 ± 3.3 2–14	0	1	1	19/19
Zhao 2021	1 device: 15	15	13	0	0	0	0	mean 5.8 ± 1.2	0	0	0	14/14
Lu 2022	1 device: 53 2 devices: 3	56	53	9	1	0	0	mean 8.3 ± 2.8 3–12	0	0	7	50/56

y, year; WCS, Willis covered stent; n, number; m, month.

### 3.3 Quality assessment

Methodological integrity of each study was assessed using the AHRQ checklist, and the details are presented in Table 3. Among them, 4 studies were of high quality and 7 were of moderate quality. The scores were between 4 and 8, and the mean was 6.6. Publication bias test was not completed as limited by the nature of noncomparative study.

**Table 3:** AHRQ checklist.

Author, y	(1)	(2)	(3)	(4)	(5)	(6)	(7)	(8)	(9)	(10)	(11)	Total
Wang2011	★	★	★	★		★	★			★	★	8
Pan2015	★	★	★	★		★	★			★		7
Liu2016	★	★				★				★		4
Kang2019	★	★	★	★		★				★		6
Liu2019	★	★	★	★		★				★		6
Deng2020	★	★	★	★		★	★			★	★	8
Liao2020	★	★	★	★		★	★			★	★	8
Wangkai2020	★	★	★	★		★				★		6
Wangwei2020	★	★	★	★		★				★		6
Zhao2021	★	★	★	★		★				★		6
Lu2022	★	★	★	★		★	★			★	★	8

AHRQ, Agency for Healthcare Research and Quality; y, year. (1) Define the source of information (survey, record review). (2) List inclusion and exclusion criteria for exposed and unexposed subjects (cases and controls) or refer to previous publications. (3) Indicate time period used for identifying patients. (4) Indicate whether or not subjects were consecutive if not population-based. (5) Indicate if evaluators of subjective components of study were masked to other aspects of the status of the participants. (6) Describe any assessments undertaken for quality assurance purposes (e.g., test/retest of primary outcome measurements). (7) Explain any patient exclusions from analysis. (8) Describe how confounding was assessed and/or controlled. (9) If applicable, explain how missing data were handled in the analysis. (10) Summarize patient response rates and completeness of data collection. (11) Clarify what follow-up, if any, was expected and the percentage of patients for which incomplete data or follow-up was obtained.

### 3.4 Outcomes of PSAs treated by WCSs

**Fig. 2:**
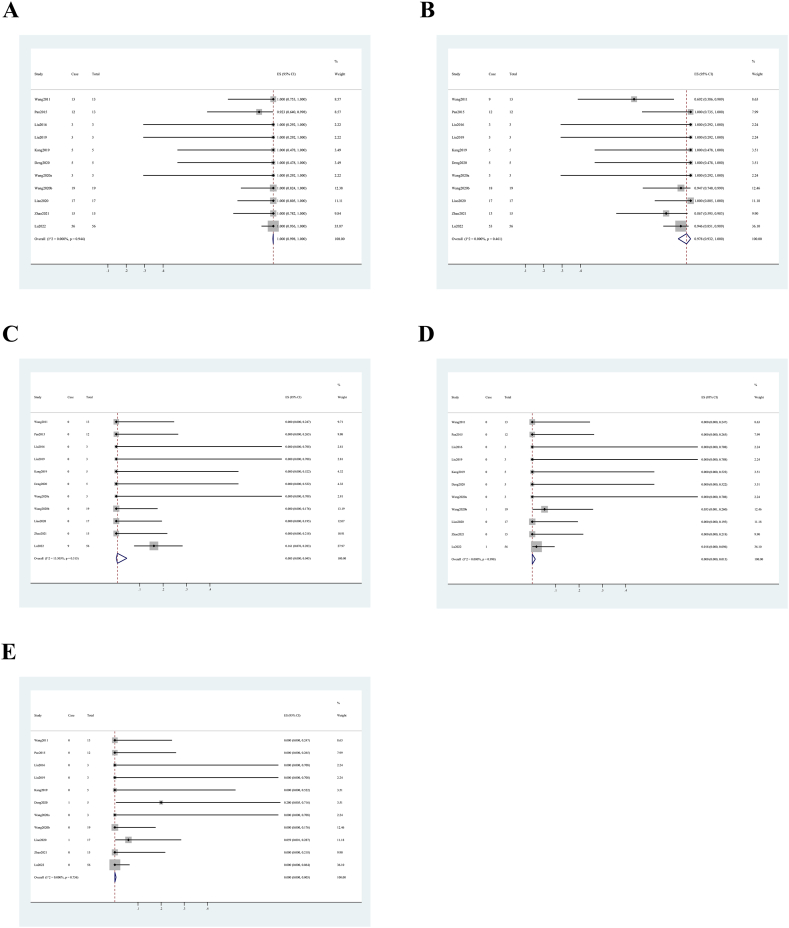
Forest plots of intraoperative outcome rate. (A) technical success. (B) complete occlusion. (C) side branch occlusion. (D) acute in-stent thrombosis. (E) intraoperative hemorrhage.

**Fig. 3:**
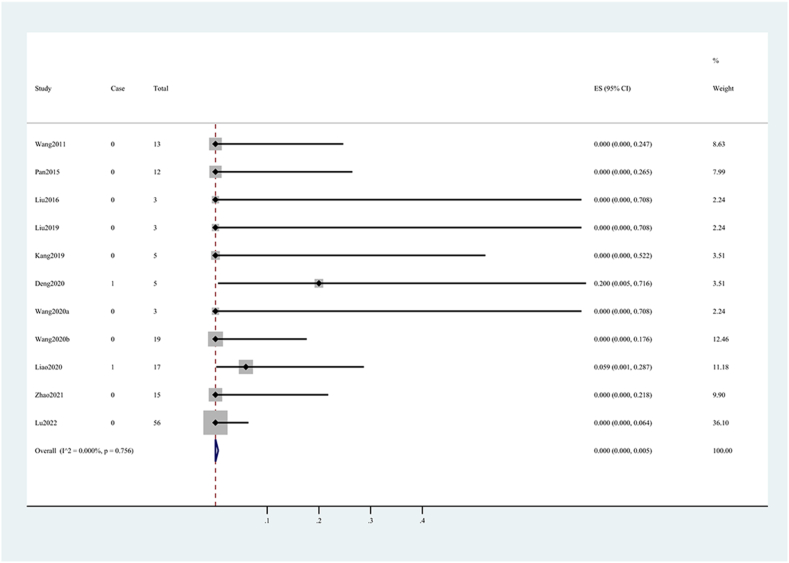
Forest plot of surgery-related mortality rate.

**Fig. 4:**
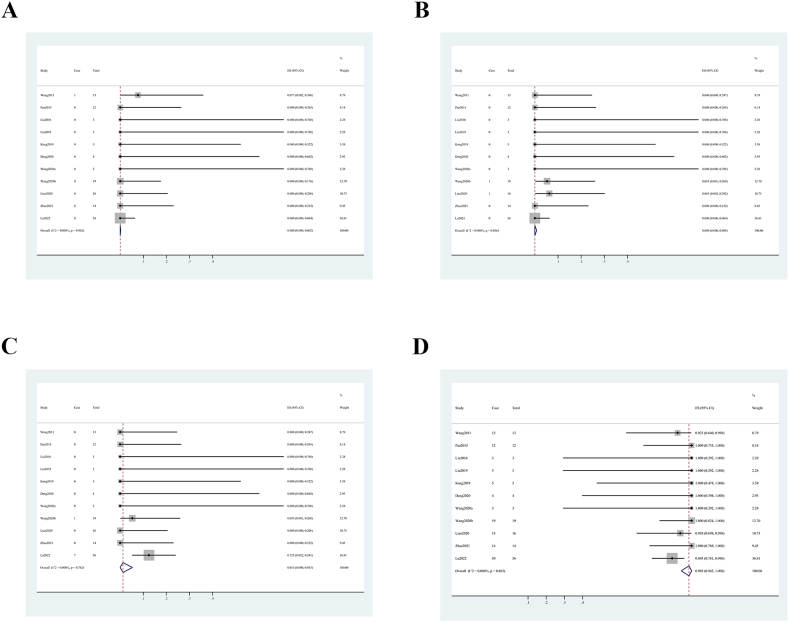
Forest plots of follow-up outcome rate. (A) recurrence. (B) parent artery stenosis. (C) late in-stent stenosis. (D) good outcome.

Technical success rate was nearly 100 % (>0.999 (95 % CI, 0.958, 1.000)) (Fig. 2A), complete occlusion rate was 97.8 % (95 % CI, 0.932, 1.000) (Fig. 2B), and side branch occlusion rate was 0.5 % (95 % CI, 0.001, 0.045) (Fig. 2C) in intraoperative situation. The rates of acute in-stent thrombosis (<0.001 (95 % CI, 0.000, 0.013)) (Fig. 2D) and hemorrhage (<0.001 (95 % CI, 0.000, 0.005)) (Fig. 2E) were both less than 0.1 %. No studies reported vasospasm or dissection. After 3–53 months follow-up, none of the included studies reported any postoperative hemorrhage or infarction events. Surgery-related mortality rate was less than 0.1 % (<0.001 (95 % CI, 0.000, 0.005)) (Fig. 3). The rates of recurrence (<0.001 (95 % CI, 0.000, 0.002)) (Fig. 4A) and parent artery stenosis (<0.001 (95 % CI, 0.000, 0.008)) (Fig. 4B) were both less than 0.1 %, while late in-stent stenosis occurred in 1.3 % (95 % CI, 0.000, 0.053) (Fig. 4C) of patients in the follow-up data. In the end, 98.5 % (95 % CI, 0.943, 1.000) (Fig. 4D) of patients had a good outcome. Sensitivity analysis was not completed as limited by the nature of noncomparative study. Table 4 provides a general summary of the overall outcomes.

**Table 4:** Overall outcomes of the meta-analysis.

Outcome	Risk Difference (95 % CI)	Raw Proportion	I [] (%)	Chi [], P value	Tau []
Technical Success	>0.999 (0.998, 1.000)	151/152	0	4.082, 0.944	0
Complete Occlusion	0.978 (0.932, 1.000)	141/151	0	9.766, 0.461	0
Side Branch Occlusion	0.005 (0.000, 0.045)	9/151	14	11.561,0.315	0.012
Acute In-stent Thrombosis	<0.001 (0.000, 0.013)	2/151	0	1.686, 0.998	0
Intraoperative Hemorrhage	<0.001 (0.000, 0.005)	2/151	0	6.672, 0.756	0
Surgery-related Mortality	<0.001 (0.000, 0.005)	2/151	0	6.672, 0.756	0
Recurrence	<0.001 (0.000, 0.002)	1/148	0	4.115, 0.942	0
Parent Artery Stenosis	<0.001 (0.000, 0.008)	2/148	0	4.920, 0.896	0
Late In-stent Stenosis	0.013 (0.000, 0.053)	8/148	0	6.607, 0.762	0
Good Outcome	0.985 (0.943, 1.000)	140/148	0	5.397, 0.863	0

CI, confidence interval; I^2^, I-squared; Chi^2^, Chi-squared; Tau^2^, Tau-squared.

## 4 Discussion

Any treatment for a rare disease would be controversial due to the difficulty of conducting randomized controlled trials and large cohort studies, and PSA was no exception. The one and only treatment for PSA before the rise of endovascular therapy was microsurgery. However, the friability, lack of true neck, and fusiform morphology of PSA made microsurgery threatening and challenging [27]. Then, endovascular therapy was more widely performed with the advances in techniques and materials. Among them, FD and covered stent attracted the most attention of surgeons and interventional radiologists [3]. A majority of patients were only found to have PSAs after they rupture due to the lack of awareness of cerebrovascular diseases among Chinese doctors and people, and this situation limited the application of FD in China. Compared with FD, covered stent could induce cavity thrombosis of PSA immediately, which enabled it to treat ruptured PSA [26].

Four different types of covered stent had been developed and documented prior to WCS, but the application was limited by their disadvantages such as delivery difficulty, size mismatch, and vessel damage by high-pressure release [24]. Li et al. designed WCS based on their considerable experience in treating intracranial complex vascular lesions in China and applied WCSs to treat PSAs of ICA for addressing the aforementioned disadvantages [28]. Since then, several clinical centers have accepted this “China Option,” and an increasing number of reports have been available on the treatment of PSA with WCS.

These clinical centers have applied WCSs to treat PSAs, but the efficacy and safety were unknown as limited by the rarity of PSA and the innovation of WCS. A higher level of evidence is required. Therefore, we pooled and analyzed all available studies to reach more reliable conclusions. Low rates of intraoperative and postoperative complications, as well as high rates of technical success and complete occlusion, were observed. Follow-up data showed that most patients ended up with a good prognosis.

Publication bias, as the only bias that can be analyzed by statistical methods, cannot be completed due to the nature of noncomparative study, which to some extent affects the stability of the results of meta-analysis of all non-controlled studies [15]. Although there was no escaping the above problems, we still used multilingual retrieval to reduce language bias, retrieved as many databases as possible to reduce database bias, and improved the search strategy to reduce search bias. What's more, selector bias, extractor bias and quality evaluation bias were controlled by discussing disagreements with a third author to reach a consensus. These relatively comprehensive methods enhanced the stability of the results of this study to a certain extent. In addition, we observed that all the included studies had no score on items 5,8 and 9 in the AHRQ checklist. That is, they didn't indicate if evaluators of subjective components of study were masked to other aspects of the status of the participants, describe how confounding was assessed and/or controlled, and explain how missing data were handled in the analysis. Although all these studies had medium-high quality according to the criteria, the influence of the above problems on the stability of the overall results cannot be ignored.

The technical success rate was nearly 100 % in our meta-analysis. Only one case reported by Pan et al. [17] failed because it had an enormous PSA of ICA and lacked a suitable WCS. The current view is that relatively poor flexibility of the stent and strong stiffness of the delivery system limit the application of WCS in the winding ICA [20]. However, the current data showed that experienced surgeons can properly handle these problems and successfully implant WCSs. Complete occlusion rate of WCS treatment, as an important factor to assess the efficacy, was 97.8 %. This rate is relatively high, but it still represents 2.2 % of patients who experience a complication that should not be ignored, that is, endoleak. Persistent endoleak may cause continuous PSA dilation or rupture by increasing the internal pressure of PSA, which is due to that PSA cavity's blood flow is unsmooth [16]. In general, balloon re-inflation at the distal and proximal parts of the stent could restrain most of immediate endoleak [26]. Zhao et al. [25] used postoperative auxiliary compression for 30 min, once or twice daily for 1–2 months, to the carotid artery on the lesion side. Overall, these patients should be followed up with more rigorous angiography. Side branch occlusion rate was 0.5 %. Nine cases of ophthalmic artery occlusion were recorded by Lu et al. [26], but none of them exhibited symptoms given that the external carotid artery system could maintain blood flow [29]. Unlike the abovementioned situation, occlusion of anterior choroidal artery or posterior communicating artery can cause catastrophic ischemic events [30,31]. This consideration reminds surgeons and interventional radiologists that they must carefully evaluate the angiogram from multiple angles to avoid side branch occlusion as much as possible. We noticed that some included studies dealt with only intracranial segment of ICA; some with only extracranial, and others with both of them. Given the differences of anatomy in these segments, such as extracranial segment of ICA does not have any branches, side branch occlusion has less potential. Therefore, the data of side branch occlusion rate were only for reference to some extent. A total of 2 of 151 patients experienced acute in-stent thrombosis. The affected arteries were recanalized successfully by injection of tirofiban, and no ischemic event was caused. According to the report of Liu et al. [20], WCSs have relatively higher thrombogenicity than other stents. Fortunately, acute in-stent thrombosis is easy to detect and treat during surgery. No studies reported vasospasm or dissection, but these complications may be caused by relatively poor flexibility of the stent and strong stiffness of the delivery system [20]. Intraoperative hemorrhage occurred in 2 of 151 patients; one was balloon rupture, and the other was distal ICA rupture. Intraoperative hemorrhage is often difficult to be predict because of its strong tendency to individuation [21]. The improvement of new materials, the gathering of experience, and the development of techniques are all essential to avoid this condition. Remarkably, the two patients died of intraoperative hemorrhage. Nevertheless, the hemorrhage and surgery-related mortality rates were lower than those of saccular aneurysm stent-assisted coil embolization [32]. In the meantime, none of the studies that were included reported any infarction events. The aforementioned data all supported the safety of 10.13039/100005997WCS therapy for PSA.

After the mean of 5.8–44.0 months of follow-up, recurrence occurred only in 1 of 148 patients. According to the description of Wang et al. [16], this patient experienced endoleak and the endoleak persisted without abating after 3 months follow-up. With a longer follow-up period, this recurrence may spontaneously resolve. Late in-stent stenosis occurred in 1.3 % of patients. A total of 2 of 148 patients experienced parent artery stenosis (the dual antiplatelet therapy was stopped for roughly 30 days following surgery in one patient). The study of Lu et al. [26] revealed that irregular antiplatelet medication was one of the strongest predictors of late in-stent stenosis. Chronic diseases including hypertension, hyperlipemia, and diabetes also increase the risk of in-stent stenosis [33]. Surprisingly, all these patients with stenosis were asymptomatic, but this complication cannot be ignored. The optimal monitoring strategy is still routine, long-term angiography follow-up. Ultimately, 98.5 % of patients had a good outcome. The aforementioned data all supported the efficacy of 10.13039/100005997WCS therapy for PSA.

Our study further confirmed the safety and efficacy of WCS treatment for PSA. Along with the simple placement procedure, short operation time, and relatively broad indications with low price, WCS has excellent generalizability and applicability in diverse patient populations and practice settings. First, it could induce cavity thrombosis of PSA immediately, which enabled it to treat both ruptured and unruptured PSA [26]. This effectively filled the technical gap that FD can only be used to treat ruptured PSA. Second, the relatively low price and good efficacy of WCS could effectively reduce the financial burden of patients, especially those in poor situations. thereby improving the treatment rate of diverse patient populations. Third, the simple placement procedure and good safety of WCS could make it generalizable and applicable in diverse practice settings [21]. Doctors in relatively low-level hospitals could also use WCS after systematic training, in this way the generalizability of WCS would be further enhanced with the increase of surgical cases.

We also have some suggestions for different stakeholder groups. For clinicians, since this study has provided a higher level of clinical evidence, when evaluating the situation and providing treatment to patients, it can be more based on comprehensive consideration, and is no longer limited to a single or fixed surgical method. For policymakers, they can cooperate with clinicians to provide appropriate science popularization and education, and try to include WCS into the medical insurance coverage, so as to improve the patients’ consultation and treatment rate. For device manufacturers, more attention should be paid to the complications behind the safety and efficacy of WCS, and WCS should be continuously optimized with the feedback of clinicians in order to achieve the best clinical use effect. At the same time, the production cost should be continuously reduced through technical means in the process, so as to reduce the price. This will allow more patients to afford to choose the most appropriate treatment.

Our research obtained positive results, but some limitations should be addressed. The rarity of PSA and the innovation of WCS cause difficulty in conducting studies of large sample size, while the selection bias is difficult to control. When performing surgical treatments with new devices, clinicians tend to choose patients who were more likely to be treated successfully in experience, which undoubtedly amplifies the safety and efficacy [34]. Some cases were ruled out with the exclusion of study during the literature search period. The reason is that not all treated lesions were PSAs or not all stents’ brands were WCSs among these works. The lack of these cases makes the selection bias much more evident. And it is not possible to assess how selection bias in this part might have affected the outcome. Besides, as far as we know, this new device is being widely promoted in China at present. It has not been promoted internationally until strong evidence is obtained (the purpose of our study was to get additional substantial proof), so there is no research in other countries for the time being. And our literature search confirmed this, all published studies were from China. In this way the selection bias may increase in the meta-analysis. This makes the results of this study only applicable to clinical centers in China for now. In addition, although all these included studies have medium-high quality according to the criteria, they all have no score on items 5,8 and 9 in the AHRQ checklist. The above situations will influence the stability of the overall results, amplify the good outcomes to some extent. Sensitivity analysis and publication bias test were not completed as limited by the nature of noncomparative study. The authenticity of our results may be affected to a certain extent.

This study is just a start in the professional field, the findings could serve as a reference for upcoming clinical trials. For ruptured PSA, application of WCS could be compared with general stent endovascular treatment. For un ruptured PSA, it could be compared with FD treatment. Clinicians should design multicenter prospective cohort studies with relatively large sample sizes to first explore the short-term efficacy and safety of WCS, and develop long-term follow-up protocols for each patient to determine long-term outcomes. At the same time, economic comparisons should not be ignored. Policymakers and device manufacturers should also be involved, providing financial, policy, and equipment support for the trials. In a word, the more robust evidence requires a multi-faceted effort.

## 5 Conclusions

WCS is a novel covered stent that has been increasingly used in several clinical centers in China. The limited evidence that exists suggests that the application of WCS in PSA treatment could be effective and safe. The improvement of new materials, the gathering of experience, and the development of techniques are all essential for the good prognosis of PSA patients. The findings of this study could serve as a reference for upcoming clinical trials.

## Data Availability statement

Data included in article/supplementary material/referenced in article.

## Funding

This study was supported by the 10.13039/501100001809National Natural Science Foundation of China (10.13039/501100001809NSFC 81870927) and the Natural Science Foundation Project of Chongqing Science and Technology Commission (CSTB2023NSCQ-MSX0112).

## Ethics statement

Review and/or approval by an ethics committee was not needed because this study is a systematic review and meta-analysis. Informed consent was not required because this study is a systematic review and meta-analysis.

## CRediT authorship contribution statement

**Jiahe Tan:** Conceptualization, Data curation, Formal analysis, Methodology, Software, Writing - original draft. **Rui Song:** Data curation, Formal analysis, Software. **Siyue Luo:** Data curation, Investigation. **Yinrui Ma:** Data curation. **Jun Su:** Data curation. **Baoping Qin:** Conceptualization, Supervision, Writing - review & editing. **Zhaohui He:** Conceptualization, Project administration, Supervision, Writing - review & editing.

## Declaration of competing interest

The authors declare that they have no known competing financial interests or personal relationships that could have appeared to influence the work reported in this paper.
